# Ultrasound–guided posterior quadratus lumborum block for postoperative pain control after minimally invasive radical prostatectomy: a randomized, double–blind, placebo–controlled trial

**DOI:** 10.17179/excli2021-4615

**Published:** 2022-01-27

**Authors:** Bartosz Horosz, Katarzyna Bialowolska, Anna Kociuba, Jakub Dobruch, Malgorzata Malec-Milewska

**Affiliations:** 1 Department of Anesthesiology and Intensive Care, Center of Postgraduate Medical Education, Orlowski Hospital, Ul. Czerniakowska 231, Warsaw, Poland; 2 Department of Urology, Center of Postgraduate Medical Education, Orlowski Hospital, Ul. Czerniakowska 231, Warsaw, Poland

**Keywords:** Quadratus lumborum block, minimally invasive prostatectomy, postoperative pain, laparoscopy

## Abstract

A minimally invasive approach to radical prostatectomy offers improved ambulation and discharge times. Postoperative pain control is one of the key factors that facilitates rapid recovery. With the aim to assure adequate analgesia and minimize the use of opioids, application of truncal nerve blocks has been proposed in a number of endoscopic procedures. The aim of this double-blind, placebo-controlled study was to evaluate the efficacy of bilateral posterior quadratus lumborum block (pQLB) in alleviating pain and reducing postoperative opioid demand in patients following endoscopic extraperitoneal and laparoscopic prostatectomy. We enrolled 50 patients who were diagnosed with prostate cancer and scheduled for prostatectomy. They were randomized to receive preoperative, ultrasound-guided pQLB with the use of either 30 ml of 0.375 % ropivacaine (ropivacaine group) or 30 ml of 0.9 % NaCl (placebo group). Our primary endpoint was opioid consumption in the first 24 hours after surgery. Secondary endpoints were pain intensity at predefined timepoints and the incidence of nausea and vomiting and pruritus. No differences were detected between the ropivacaine and placebo groups in intravenous oxycodone consumption during the first 24 hours after surgery. Similarly, there were no differences in pain intensity at any of the timepoints assessed. The rate of nausea and vomiting was equal in both groups and pruritus was not observed. Application of bilateral pQLB does not reduce opioid consumption after minimally invasive prostatectomy.

## Introduction

In recent years, minimally invasive techniques have gained popularity in the surgical treatment of prostate cancer. Laparoscopic radical prostatectomy (LRP) and robotic-assisted laparoscopic radical prostatectomy (RALP) improve postoperative recovery and ambulation with preserved oncological outcomes (Basiri, 2018[3]). Despite being less invasive surgical interventions, laparoscopic procedures may still be demanding in terms of postoperative pain, because both somatic and visceral pain pathways are involved. Regional anesthesia techniques have been utilized to alleviate the pain and to optimize enhanced recovery after laparoscopic prostatectomy, with the aim to avoid or decrease the need for opioids (Bae et al., 2017[2]; Cacciamani et al., 2019[5]; Shahait and Lee, 2019[21]; Taha et al., 2019[22]). Truncal blocks as an addition to local wound infiltration have been used with promising outcomes and have proved to be superior to wound infiltration alone (Cacciamani et al., 2019[5]; Taha et al., 2019[22]). Transversus abdominis plane (TAP) block is currently recommended for laparoscopic and robotic prostatectomy by the recent procedure specific postoperative pain management (PROSPECT) guidelines (Lemoine et al., 2021[17]).

Although effective in alleviating abdominal wall pain, these techniques cannot block the visceral component of pain. The quadratus lumborum block (QLB) technique, first described in 2007 by Blanco as the most posterior modification of TAP block, is said to be more potent in blocking somatic pain, with the option to reduce visceral pain, due to the spread of local anesthetic towards the paravertebral space and a numbing effect on the sympathetic trunk (Akerman et al., 2018[1]; Elsharkawy et al., 2019[10]; Tamura et al., 2019[23]). The use of QLB as a part of postoperative multimodal analgesia following minimally invasive prostatectomy has been described but has not been validated in a randomized, placebo-controlled trial (Theisen and Davies, 2020[24]).

In this study, we aimed to investigate the effect of bilateral, ultrasound-guided posterior quadratus lumborum block (pQLB) on postoperative opioid consumption and pain control after laparoscopic or extraperitoneal endoscopic radical prostatectomy (LRP or EERP, respectively).

## Methods

**Figure 1:**
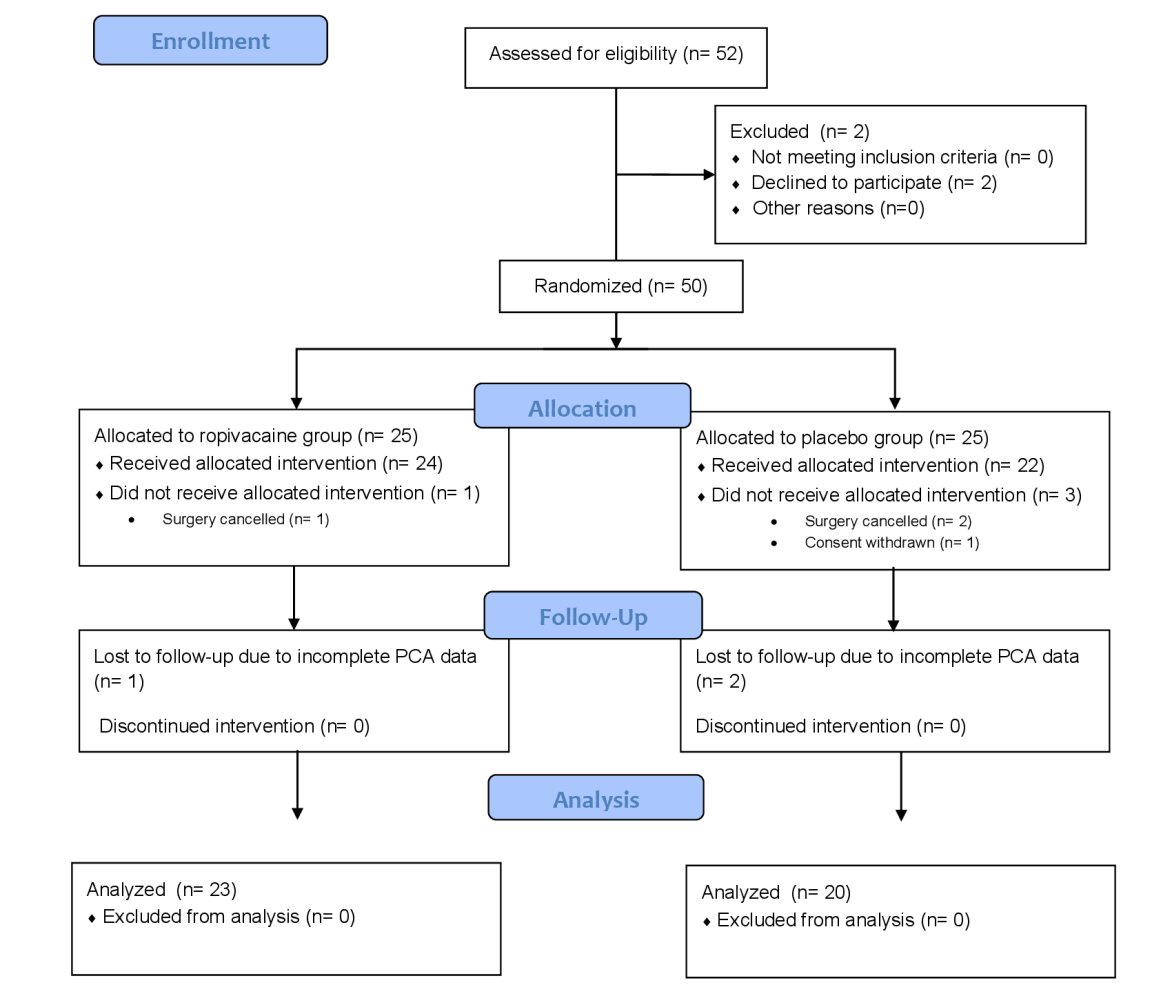
Consolidated Standards of Reporting Trials (CONSORT) flow diagram. PCA, patient-controlled analgesia

The study protocol was approved by the institutional Ethics Committee on 4 July 2018 (83/PB/2018) and performed at the Department of Anesthesiology and the Department of Urology, Orlowski Hospital, Center of Postgraduate Medical Education, Warsaw, Poland. It is reported in accordance with the Consolidated Standards of Reporting Trials (CONSORT) guidelines on reporting parallel group randomized trials and was registered at ClinicalTrials.com (NCT03600129). The CONSORT flow diagram is shown in Figure 1(Fig. 1). Patients scheduled to undergo either LRP or EERP for prostate cancer were to be enrolled. The inclusion criteria were: age 40-80 years and American Society of Anesthesiologists (ASA) physical status ≤ 3. Patients allergic to local anesthetics, with infection in the area of planned injection site and patients experiencing chronic pain were excluded from the study, as well as patients with significant communication disorders and those unable to understand the principle and apply the rules of intravenous patient-controlled analgesia (PCA). 

### Patients and pre-specified endpoints

Fifty patients agreed to participate in the study and were enrolled between July 2018 and October 2019 after signing the informed consent form. Online randomization with a 1:1 allocation ratio was performed by using GraphPad QuickCalcs randomizer (https://www.graphpad.com/quickcalcs/randMenu/) for 50 subjects. Before the surgery, the sequentially numbered, sealed, opaque envelope containing the group allocation and a description of the solution to be used for the block placement was handed to an anesthetist not involved in the perioperative care of the patient enrolled in the study. A total of 60 ml of study solution was prepared for each patient: 2 × 30 ml of 0.375 % ropivacaine in the ropivacaine group or 2 × 30 ml of 0.9 % sodium chloride (saline) in the placebo group. All syringes were labelled in the same way and handed to anesthetist responsible for conducting the anesthesia and block placement, who was blinded to the group allocation. The primary endpoint of the study was oxycodone consumption in the first 24 hours after surgery.

The secondary endpoints were pain intensity at 1, 6, 12 and 24 hours after surgery and the incidence of opioid-related complications: nausea and vomiting and pruritus.

### Block placement and intraoperative management

Patients received 7.5 mg oral midazolam 1 hour prior to induction of anesthesia. After admission to the operating room, routine monitoring was applied (non-invasive blood pressure, three-lead electrocardiogram monitoring and pulse oximetry). Once intravenous access was secured with an 18G cannula, slow infusion of balanced crystalloid was initiated and 1 g of paracetamol and 4 mg of dexamethasone was given intravenously. Then, general anesthesia was induced with 100 µg of fentanyl and 2-2.5 mg/kg of propofol. Rocuronium 0.6 mg/kg was used for muscle paralysis. Anesthesia was maintained with sevoflurane in the oxygen/air mixture, with the use of repeated boluses of 100 µg of fentanyl as required for hemodynamic stability, at the discretion of the anesthetist who was responsible for the whole procedure.

After induction of general anesthesia and before surgical draping, bilateral pQLB under ultrasound guidance was performed. All blocks were placed with patient in the supine position, with a contralateral tilt to facilitate sufficient ultrasound visualization of the desired posterolateral area of the abdominal wall. After thorough disinfection of the area and maintaining sterile conditions throughout the procedure, the linear ultrasound probe (Phillips Sparq, Phillips Polska LLC, Warsaw, Poland) was placed in the transverse plane above the anterior superior iliac spine and moved cranially until typical layers of the external oblique (EO), internal oblique (IO) and transversus abdominis (TA) muscles were visualized. Then, the probe was moved laterally and posteriorly to visualize and trace the muscular layers of the abdominal wall. Once the posterolateral border of TA was identified, the probe position was adjusted to visualize the hyperechoic middle layer of the thoracolumbar fascia on the posterior surface of quadratus lumborum (QL) muscle, which extends from the posterior aponeurosis of the TA and IO. Using the in-plane technique, a 22G spinal needle was introduced in the lateroposterior direction and advanced until it reached the posterior surface of the QL, at which point a distinct 'pop' is usually felt, indicating that the needle tip has entered the fascia. The position of the needle tip was confirmed with hydrodissection. In the ropivacaine group, 30 ml of 0.375 % ropivacaine was injected after negative aspiration for blood, whereas in the placebo group, 30 ml of 0.9 % sodium chloride was injected in the same manner. The deposition of the injectate in the desired space was observed and confirmed with ultrasound. The procedure was repeated on the other side. 

pQLB was either placed by an anesthetist with > 5 years of experience in peripheral nerve blocks and regional anesthesia (BH or KB) or an anesthesia trainee with at least 2 years of experience, in which case the whole procedure was supervised hands-on and the infusion of the injectate started only after the target needle position was approved by the aforementioned anesthetists. 

During the surgery, the patient was placed in the supine and steep Trendelenburg position with shoulder support and marked pelvis protrusion. In each case, five-port surgical access was established through minor skin incisions and the specific setup of the ports did not differ between the two types of surgery. Throughout both intraperitoneal and extraperitoneal procedures, the insufflation pressure did not exceed 12 mm/Hg except for an occasional increase to 20 mm/Hg to aid Santorini plexus hemostasis, as required. In contrast to EERP, LRP involved extended removal of pelvic lymph nodes as described previously (Dobruch et al., 2014[9]). Beside lymphadenectomy, radical removal of the prostate gland followed the same basic surgical and oncological principles established in patients with prostate cancer subjected to EERP and LRP. The specimen was extracted in an endo-bag through an extended infraumbilical incision previously used for trocar placement.

Once the surgery reached the stage of vesicourethral anastomosis, 2 g of metamizole and 8 mg of ondansetron were given. After trocars were removed and the operating table was returned to its neutral position, the neuromuscular blockade was reversed with neostigmine to facilitate the extubation of the trachea. The intraoperative data recorded for the purpose of the study were duration of surgery and anesthesia, estimated blood loss and the total intraoperative dose of fentanyl.

### Postoperative pain management

After surgery, the patient was moved to the post-anesthesia care unit (PACU). Oxycodone in an intravenous patient-controlled analgesia (IV-PCA) pump (Medima Medical, Warsaw, Poland) was prepared and connected to the patient's intravenous line. Routine postoperative analgesia was administered intravenously and consisted of 1 g of paracetamol and metamizole every 6 hours and 50 mg of dexketoprofen every 8 hours. The PCA pump was set to deliver 2 mg boluses with a 5 minute lock-out time and a maximum dose of 10 mg of oxycodone in 1 hour, with no background infusion. The method of using PCA was explained to patients prior to enrolment. The dose of oxycodone delivered in the assessed time intervals was derived from the PCA pump's internal memory, while the record of any additional pain intervention was extracted from PACU nursing records. Pain intensity was assessed by using the Numeric Rating Scale (NRS) at the predefined time intervals and every time the patient complained of pain. Episodes of nausea, vomiting and pruritus were noted and recorded. 

### Statistical analysis

The routine mode of postoperative oxycodone administration at our institution is 5 mg by subcutaneous injection given in 3-hour intervals, with intravenous boluses of 2 mg for breakthrough pain. Sample size calculation was based on the results of the retrospective analysis of PACU charts of minimally invasive prostatectomy cases, which revealed that the average of two doses (10 mg) was usually required in the first 24 hours after surgery. As we assumed at least a 25 % reduction in opioid requirement to be of clinical significance in this case, the number of required cases was 16 in each group for the test to achieve 80 % power at the level of significance of 0.05. Randomization was performed for 50 cases to allow for a number of dropouts. The data were assessed for normality by using the Shapiro-Wilk test. An unpaired Student's t-test was used to compare normally distributed data and the Mann-Whitney U-test was used to compare continuous data with a non-normal distribution. Continuous data are presented as mean ± standard deviation (SD) or median [interquartile range (IQR)]. For comparison of dichotomous variables, Fisher's exact test was employed, and the data are presented as number (%). The analyses were performed with R version 3.5.1 (http://cran.r-pro-ject.org).

## Results

Fifty patients were enrolled between July 2018 and October 2019, with 25 in each group. Twenty-four patients in the ropivacaine group received pQLB, as in one case the surgery was cancelled on the day of surgery due to the sudden onset of cardiac symptoms. In the placebo group, out of 25 enrolled patients, 22 received pQLB: one patient decided to withdraw his consent to participate in the study and two cases were rescheduled due to technical issues. Complete data were available from 23 cases in the ropivacaine group and 20 cases in the placebo group, due to erroneous cessation of postoperative PCA treatment (1 and 2 cases, respectively). 

Patient demographics and surgery-related data are presented in Table 1(Tab. 1). The ropivacaine and placebo groups were not different in terms of demographics and perioperative data. The median length of hospital stay was short and similar between the groups.

The total oxycodone consumption in 24 hours was not different between the patients in the ropivacaine and placebo groups. Postoperative data are given in Table 2(Tab. 2). Similarly, no differences were found between postoperative oxycodone consumption when the assessed time intervals were considered: the first 6 hours, 6-12 hours and 12-24 hours after completion of surgery. The highest demand for opioids was found during the first 6 hours, while after 12 hours the need for PCA-delivered oxycodone was negligible in both groups.

The NRS was similar in the ropivacaine and placebo groups. No differences were found in pain levels at rest at 1, 6, 12 and 24 hours after surgery, as well as during a cough 24 hours after the surgery. The maximum NRS 1 hour following surgery was 7 in the placebo group and 8 in the ropivacaine group (data not shown). The incidence of nausea and vomiting was low and similar in both groups; pruritus was not noted.

See also the Supplementary data.

## Discussion

This study found no differences in postoperative opioid consumption and pain intensity in patients undergoing minimally invasive prostatectomy and given pQLB compared with patients given the sham block. Using a double-blind, placebo-controlled study design, we were not able to detect the expected differences in the primary and secondary endpoints. 

Ultrasound-guided pQLB is described as a safe and straightforward technique, as the point of local anesthetic deposition is relatively superficial and separated from the abdominal cavity by the QL muscle, minimizing the risk of intraperitoneal injection and bowel injury. In most cases, the procedure can be performed easily in the supine position, which reduces the time needed to place the block and makes it convenient to be performed after induction of general anesthesia. It results in the anesthesia of the nerve fibres innervating the anterior and lateral abdominal wall (Elsharkawy et al., 2017[11]). Moreover, with the prospect of local anesthetic reaching the paravertebral space, this technique is said to be able to provide more effective analgesia than TAP block for abdominal surgery. The use of QLB for postoperative pain control has been proposed and validated in a number of surgical procedures, with promising results (Elsharkawy et al., 2019[10]). 

Endoscopic techniques in urology have developed to the point where most of the major procedures are performed using a minimally invasive approach (Eswara and Ko, 2019[12]; Fantus et al., 2019[13]). Despite less extensive surgical trauma, postoperative pain control may still pose a challenge and require administration of potent opioids (D'Alonzo et al., 2009[7]; Remzi et al., 2005[19]; Webster et al., 2005[25]; Woldu et al., 2014[26]). Therefore, the use of peripheral nerve blocks is warranted, with the goal of alleviating pain and reducing the incidence of opioid-related complications.

In the retrospective, comparative analysis of 200 patients who underwent RALP, Rogers et al. reported a significant reduction in both pain intensity and narcotic use, as well as a reduced time to ambulation when TAP block was utilized as part of the pain control plan (Rogers et al., 2021[20]).

In their randomized and blinded study, Dal Moro et al. described an improvement in pain control and demand for analgesics in RALP after administration of TAP block. The use of four injections of 50 ml of 0.25 % levobupivacaine each was substantially effective in alleviating the pain throughout the entire 24-hour postoperative period (Dal Moro et al., 2019[6]). 

Similar findings have been reported after the use of QLB in other urologic procedures. In their placebo-controlled study, Dam et al. showed a significant reduction in the opioid requirement during the first 12 hours after surgery and shorter hospital stay after percutaneous nephrolithotomy when unilateral transmuscular quadratus lumborum block (TQLB) was employed (Dam et al., 2019[8]). In a similarly designed trial, Kwak et al. (2020[16]) investigated the effect of one-sided lateral QLB in laparoscopic nephrectomy. Their trial demonstrated both a significant reduction in opioid consumption and pain intensity in the intervention group compared with patients given the sham block.

Surprisingly, our results failed to reproduce the above findings with the use of pQLB for LRP and EERP. Despite the different approach, both of these procedures involve multiple skin incisions for port insertion and extensive extraperitoneal manipulation in the lower abdomen and pelvis, which concludes with vesicourethral anastomosis. Therefore, the resulting somatic pain is primarily conducted through the fibres of the low thoracic and high lumbar segments, while visceral pain is mediated by the sympathetic trunk and parasympathetic pathways.

In our opinion, the most likely explanation for the lack of differences in pain intensity after the block performed with local anesthetic and placebo in our patients is a specific distribution of anesthesia after pQLB. Despite the spread of local anesthetic to the paravertebral space and numbing of the thoracic sympathetic trunk, the level of anesthesia appears not to be sufficient for the extent of extraperitoneal pelvic surgery. The visceral component of pain derived from the surgical trauma to the pelvic floor, the bladder and the proximal urethra is likely to play a significant role. It is conveyed via the parasympathetic (S2-S4) and sympathetic (T10-L2) fibres that form the pelvic plexus. Moreover, animal studies have shown that the parasympathetic component is likely to be dominant (Origoni et al., 2014[18]). Hence, it may be argued that the inability to affect the pelvic parasympathetic pathways could be responsible for our results. This could also explain the contrast between our findings and the results of aforementioned studies regarding laparoscopic nephrectomy and percutaneous nephrolithotomy (Dam et al., 2019[8]; Kwak et al., 2020[16]), as the component of visceral pain in minimally invasive prostatectomy is obviously much more prominent.

Similarly, a different characteristic of pain distribution may be the reason for which our results did not reproduce the outcomes of studies where TAP was used for RALP (Dal Moro et al., 2019[6]; Rogers et al., 2021[20]). The higher number and size of the trocars used for RALP when compared to LRP and EERP results in a greater injury of the abdominal wall. Therefore the contribution of somatic pain may be more pronounced and pain relief that is achieved with TAP more significant.

Our findings are in line with the results of the recent study by Hansen et al. (2021[14]): they found no differences in opioid use and pain intensity after laparoscopic hysterectomy in patients given TQLB with either local anesthetic or placebo. The authors speculated that the inability to affect the parasympathetic component of the uterovaginal plexus was responsible for the lack of TQLB effect. Their explanation may be supported by the results of the study by Ishio et al. (2017[15]), who reported a marked benefit of bilateral, posterior QLB in patients undergoing less extensive laparoscopic gynecologic surgery. 

There are a few limitations of our study. First, to maintain the double-blind assessment, the analgesic effect of the block was not assessed at any point of the study. Second, it can be argued that pain related to extraperitoneal and laparoscopic procedures may differ and thus these cases should not be analyzed together. Third, the sample size calculation was based on retrospective data regarding intermittent, 'when required' dosing of subcutaneous opioids. A different mode and route of opioid administration (PCA) used in the study could have resulted in a different effective dose. Fourth, the fact that all cases were performed by a surgeon (DJ) with extensive experience in LRP and EERP (more than 1,000 procedures) could affect the postoperative pain levels. Therefore, our results may not be representative for all minimally invasive prostatectomy procedures.

## Conclusion

Our results suggest that ultrasound-guided pQLB does not reduce the postoperative opioid requirement after LRP and EERP.

## Declaration

### Conflict of interest

The authors declare that they have no conflict of interest.

## Supplementary Material

Supplementar data

## Figures and Tables

**Table 1:**
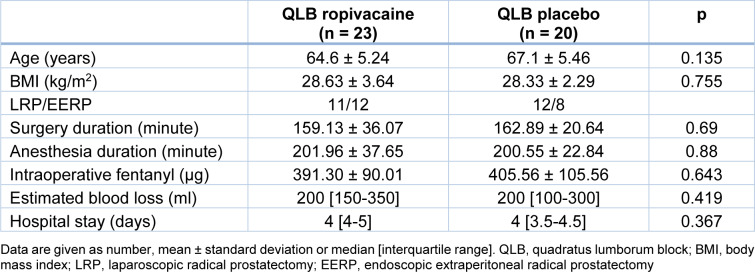
Demographics and perioperative data

**Table 2:**
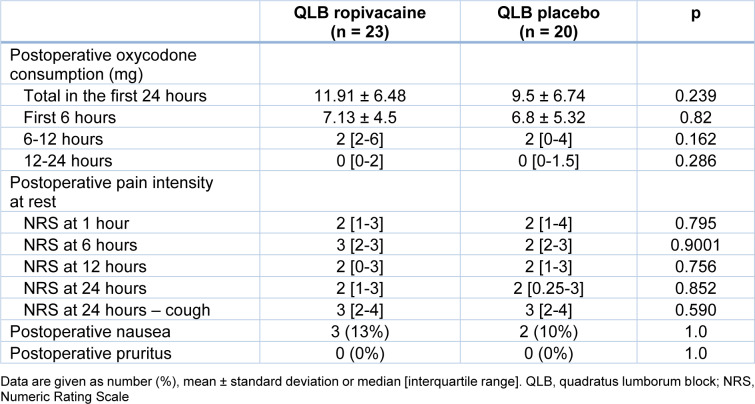
Postoperative data
